# A study of impact of climate change on the U.S. stock market as exemplified by the NASDAQ 100 index constituents

**DOI:** 10.1038/s41598-024-66109-7

**Published:** 2024-07-05

**Authors:** Cunpu Li, Yingjun Liu, Lishuo Pan

**Affiliations:** School of Economics and Finance, Xi’an International Studies University, Guodu Street, Xi’an, 710128 Shanxi Province China

**Keywords:** NASDAQ 100 index; climate change; event study methodology, Climate sciences, Climate-change impacts, Environmental economics

## Abstract

This paper employs an innovative event study methodology to demonstrate the impact of climate change on the NASDAQ index from the unique perspective of extreme weather events. This is achieved through the application of the event study methodology to a total of 526 biological, climatic, geological, hydrological, and meteorological disasters of climate change occurring in the U.S. during the period of 2000–2019. The results of the study demonstrate that: ① it can be generally observed that the five dimensions of climate change have a significant impact on stock returns. ② Empirical evidence indicates that the impact of different climate change dimensions on the return rate of stocks from NASDAQ stocks varies. In contrast, the biological and hydrological dimensions have a significantly negative impact on the return rate of stocks from the NASDAQ index, while the climate dimension has a significantly positive impact on the return rate of stocks from the NASDAQ index. ③ From the perspective of time, the impact of the five dimensions of climate change on the stock yield exhibits certain non-linear characteristics. This can be observed in the phenomenon of shock reversal, which occurs before and after the event.

## Introduction

The vulnerability of the financial system to exogenous shocks has become an established academic consensus. Extreme shock events such as war have been demonstrated to significantly affect equity volatility^1^. As a stochastic and exogenous negative event, climate change and the financial risks it triggers represent a significant cause of volatility in financial markets. The frequency of extreme weather events has doubled since 1950. The most significant consequence of climate change is global warming, which is responsible for a number of other effects, including sea level rise, extreme precipitation and extreme temperature events, hurricanes, landslides and even microbial growth. These have a detrimental impact on the economic society and financial stability^2^. In 2021, for the first time, the U.S. Financial Stability Oversight Council (FSOC) included climate change in its list of major threats affecting financial stability, as detailed in the Council’s Climate Financial Risk Report. The U.S. Secretary of the Treasury observed that the occurrence of frequent climate change catastrophes would continue to have a detrimental impact on the U.S. financial system in the future. The U.S. stock market plays a significant role in the U.S. financial market, serving as a vital instrument for diversifying and mitigating exogenous shocks. Additionally, it serves as a crucial indicator for gauging the impact of negative events on the financial system. Consequently, the assessment of the impact of climate change on the U.S. stock market will assist U.S. financial regulators, businesses and investors in identifying and mitigating the financial risks posed by climate change and maintaining the stable operation of the U.S. financial system. Based on the discussion above, this paper analyses the impact of the five dimensions of climate change on the US stock market and its patterns of change in the short and long term.

Paolo Pagnottoni et al. (2022) examined the impact of biological, climatic, geological, hydrological and meteorological disasters in 104 countries on 27 global stock market indices and found that the climatic and biological dimensions had the strongest impact on international financial markets^3^. Worthington et al. (2004) studied 42 natural disaster events in Australia and found that mountain fires, tornadoes and earthquakes had a significant negative impact on stock returns^4^. A study by Chen Zhaoweiping (2019) shows that geological disasters have a significant negative impact on stock returns in southwest and northeast China, while meteorological disasters have the opposite effect^5^. Ihtisham A. Malik et al. (2020) demonstrated that the response in the pre-disaster period is slower than in the post-disaster period^6^. Ji Qiang et al. (2022) used crawler technology to collect relevant data from listed companies in China to construct a climate risk perception index and quantitatively analyze its impact on China’s stock market^7^, and found that the impact of climate risk on China’s stock market changes over time. Ye Liping (2022) found that climate news risk has a time-varying impact on financial market uncertainty^8^ based on the wall street climate news index constructed by Robert F Engle et al^9^.

With regard to the five dimensions of climate change, the majority of studies focusing on the biological dimension utilise China as a case study to examine the impact of stock volatility during the SARS and COVID-19 outbreaks. Tong et al. (2003) pointed out that SARS pneumonia had only a short-term impact on China’s economy, and the shock caused stock prices to fall and fluctuate with the epidemic notification. Barro et al. (2020), demonstrated that the high level of lethality of influenza greatly reduced the returns on stocks and bonds^10^. Liu Haiyue et al. (2020) studied the short-term impact of COVID-19 on 21 major stock indices and found that stock markets plunged dramatically after the virus outbreak, with negative abnormal returns being more severe in Asian countries^11^. Chen Lin and Qu Xiaohui (2020) investigated the impact of COVID-19 on Shanghai and Shenzhen stock returns using a panel data fixed effects model. The study shows that there is a negative impact on the stock prices of Shanghai and Shenzhen companies^12^. Shao Changan et al. (2023) find that the negative impact of COVID-19 on shock returns in Shanghai exceeds 20 days and has some persistence^13^.

The geological dimension has been studied in a variety of regions. Liwei Shan et al. (2012) took the Wenchuan earthquake as a research object, and his study shows that in the 12 months after the earthquake, the stock returns of firms headquartered near the epicentre were significantly lower than those further away from the epicentre, and this pattern of stock returns did not exist for a long period before or after the earthquake. Hsien-Yi Lee et al. (2007) investigated the contagion effect of the Japanese stock market on the whole financial market after the strong earthquake in Southeast Asia on 26 December 2004 by using heteroskedasticity bias based on correlation coefficients^14^. Bert Scholtens et al. (2020) apply the event study methodology by using a CAPM model to solve for 87 event sample cumulative abnormal returns to analyse the impact of natural disasters on the stock market value of fishing companies^15^.

The climatic and hydrological dimensions have been relatively little studied. Ruohan Wu (2021) found that climatic and hydrological disasters have “negative–positive–positive” effects on the structural transformation of the US economy^16^. Li Min (2019) uses multiple linear regression analysis and grey prediction to find that the increase in precipitation has a positive impact on the overall operation of the financial industry, positively promoting the insurance and banking industries, but not conducive to the stable development of the securities industry^17^. The meteorological dimension has been studied extensively and in detail in all parts of the financial system. Ewing et al. (2006) find a negative impact of Hurricane Floyd on the stock prices of insurance companies^18,19^. In a way, we find that the market reacts significantly to news about the path and intensity of the storm prior to landfall, suggesting that the market is finding information.

However, Andrew Worthington (2008) used a GARCH-Mean model to investigate the impact of natural events in Australia on its stock returns. The results show that at the market level, neither natural events nor disasters have a significant impact on returns^20^.

The majority of existing climate change research focuses on both temperature and precipitation in order to assess their long-term impacts on financial markets. However, there is some scope for research on the short-term effects of climate change events. The event study approach can isolate and assess the impact of a particular event on asset prices, accurately capturing the direct effect of price changes before and after the event. This provides a more accurate understanding of the actual impact of the event on the market. The event study approach typically employs a shorter time window than long-term research methods, which enables the immediate effects of an event to be rapidly assessed. Consequently, this paper further employs the event study approach to investigate the short-term economic consequences of climate change.

### Theoretical mechanism

The risk of climate change exerts an influence on the stock price fluctuations of companies in different industries and different regions. This is achieved through a number of channels, including financial, investor sentiment and transportation trade^21^. Ultimately, this affects the stock yield.

At the organisational level, on the one hand, climate change events can result in direct and indirect losses in the immediate aftermath, which, in turn, can have a detrimental impact on business operations. In accordance with the theoretical framework proposed by Batten^21^, direct losses encompass damage to fixed assets, inventories, raw materials, extractable natural resources, and physical infrastructure (e.g., residential housing, roads, telecommunications, and electricity networks) caused by climate change events such as extreme weather, which can directly lead to negative impacts on a firm's production activities and a reduction in total factor productivity, which can deteriorate the balance sheets of listed firms in the short term. Such events can also affect the valuation of the affected firms. Indirect losses include the cost of business interruptions due to shortages of water or electricity supply caused by natural disasters, as well as alternative inputs for the repair and reconstruction of infrastructure and capital losses. These factors can lead to a reduction in investment in technological innovation, which in turn adversely affects the long-term development of the enterprise and consequently its stock price. On the other hand, the occurrence of adverse weather conditions renders commodities and transport vehicles more susceptible to damage during transit, thereby leading to direct economic losses for commercial companies and elevated insurance claims risks for insurance companies in the stock market. Extreme weather events cause disruptions in the operation of transport systems such as port shipping, airports, and railways, affecting cross-border commodity flows^22^. This, in turn, leads to higher operating costs for firms. Oh^23^ suggested that climate change significantly increases the costs of cross-border merchandise trade. For example, extreme weather may lead to the forced closure of some shipping routes, increasing the distance between air transport and liner shipping, and raising costs such as time and fuel for cross-border merchandise transport.

From the perspective of the investor, climate change events, as exogenous shocks with a high degree of uncertainty, can alter the risk appetite of investors in financial markets^24^. Firms face higher business risks in climate shocks and are forced to resort to layoffs or wage cuts to maintain operations. Once this happens, lower incomes and higher unemployment among the population are inevitable, leading to weaker expectations of households and financial institutions. As a result, investors will be more inclined to hold low-risk financial assets, such as government bonds, instead^25^. Concurrently, investors are privy to the potential impact of climate change on companies, which may result in capital chain rupture or fixed assets damage. In the context of asset value, investors may exhibit panic selling behaviour, leading to a decline in stock prices and market expectations^26^. This, in turn, affects the stock itself, resulting in lower stock yields.

## Results

### Benchmark results

The five dimensions of climate change events are summarised here: six biological catastrophes, 85 climatic catastrophes, 10 geological catastrophes, 127 hydrological catastrophes and 33 meteorological catastrophes. Climate change is a gradual process that occurs over an extended period of time. Periodic changes in climate characteristics typically persist for at least 10 years. This paper divided the dataset into two parts in 2010. This approach enabled the long-term impact of climate change to be observed and a comparative analysis to be conducted, thereby facilitating the identification of the long-term trend of climate change and the influence of time-level heterogeneity. The biological events are missing in the 2010–2019 part, and only stock returns from 2000 to 2009 are examined. The study results are shown in Table 1.

**Table 1:** Significance test results of climate change.

Climate change	2000–2009			2010–2019		
	MCAR	t	P	MCAR	t	P
Biological dimensions	− 0.004795	− 7.4040	0.0000***	/	/	/
Climatic dimensions	0.0004113	1.3759	0.0845*	0.0040697	18.1241	0.0000***
Geological dimensions	− 0.000394	− 1.4155	0.0785*	0.0002942	1.8286	0.0337**
Hydrogical dimensions	− 0.0110883	− 6.2286	0.0000***	− 0.0056124	− 4.0168	0.0000***
Meteorological dimensions	0.009523	2.3937	0.0085***	− 0.004227	− 3.4525	0.0003***

*P < 0.1.

**P < 0.05.

***P < 0.01.

Table 1 shows that the average cumulative excess return values of the sample stocks show different directions of volatility during the [− 5, 5] time window of the climate change event and different degrees of response during the two phases before and after 2010. The biological dimension of climate change rejects the initial hypothesis at the 99% confidence level, indicating that the biological dimension of climate change causes short-term volatility in US stock market returns, and a negative value of indicates a negative shock to US stock returns. The climatic dimension of climate change rejects the original hypothesis at the 90% confidence level for the period 2000–2009, indicating that the climatic dimension of climate change causes short-term volatility in US stock market returns; for the period 2010–2019, the climatic dimension rejects the original hypothesis at the 99% confidence level, indicating that climate change events in this dimension cause an increasing and highly variable shock to the US stock market, and the change is dramatic. As shown by the average cumulative excess return value, climate events have a positive effect on the return of US listed stocks over the 20 year period. The geological dimension of climate change rejects the original hypothesis at the 90% confidence level for the period 2000–2009 and at the 95% confidence level for the period 2010–2019, reflecting the increasing sensitivity of US stock market returns to events in the geological dimension of climate change and the negative effect on US stock market returns. Both the hydrological and meteorological dimensions of climate change reject the initial hypothesis at the 99% confidence level over the 20 year period, suggesting that there are strong and persistent shocks to US stock market returns from these dimensions of climate change. As shown by the average cumulative excess return values, hydrological events have a negative effect on the stock returns of US listed companies over the 20 year period, while the effect of meteorological events on the stock returns of US listed companies shifts from a positive effect in 2000–2009 to a negative effect in 2010–2019. From Table 2, it can be concluded that climate change has an impact on the US stock market.

**Table 2:** Significance test results of biological dimension.

Time window	2000–2009		2010–2019	
	MCAR	P	MCAR	P
[− 10, − 1]	0.0009289	0.0210**	/	/
[− 5, − 1]	0.0041129	0.0000***	/	/
[− 3, − 1]	0.002553	0.0000***	/	/
[1, 3]	− 0.0013426	0.0000***	/	/
[1, 5]	− 0.0032238	0.0000***	/	/
[1, 10]	− 0.0052573	0.0000***	/	/
[1, 20]	− 0.0061569	0.0000***	/	/

*P < 0.1.

**P < 0.05.

***P < 0.01.

### Multi-time-window results

In order to investigate whether the prediction, occurrence and persistence of climate change will have a time-varying effect on the stock returns of US-listed companies, this paper divides the time windows into [− 10, − 1], [− 5, − 1], [− 3, − 1], [1, 3], [1, 5], [1, 10], [1, 20] and conducts the significance tests on, respectively. Among them, [− 10, − 1] represents the long-term expected effect, [− 5, − 1] represents the medium-term expected effect, [− 3, − 1] represents the short-term expected effect, [1, 3] represents the short-term shock effect, [1, 5] represents the medium-term shock effect, [1, 10] represents the long-term shock effect and [1, 20] represents the continuous effect. The results of the significance test of before and after disaster events of the five dimensions of climate change are shown in Tables 2, 3, 4, 5, 6.

**Table 3:** Significance test results of climatic dimension.

Time window	2000–2009		2010–2019	
	MCAR	P	MCAR	P
[− 10, − 1]	− 0.0004837	0.1353	0.0033452	0.0000***
[− 5, − 1]	− 0.0002107	0.2069	0.003335	0.0000***
[− 3, − 1]	0.0009884	0.0000***	0.0018124	0.0000***
[1, 3]	− 0.0003102	0.0147**	0.000136	0.1500
[1, 5]	0.0005766	0.0009***	0.001048	0.0000***
[1, 10]	0.000507	0.0245**	0.0011205	0.0000***
[1, 20]	− 0.0008681	0.0109**	0.00143	0.0000***

*P < 0.1.

**P < 0.05.

***P < 0.01.

**Table 4:** Significance test results of geological dimension.

Time window	2000–2009		2010–2019	
	MCAR	P	MCAR	P
[− 10, − 1]	− 0.0030802	1.0000	− 0.0016048	1.0000
[− 5, − 1]	− 0.0017486	1.0000	0.0008486	1.0000
[− 3, − 1]	− 0.0002372	0.9609	− 0.0007282	1.0000
[1, 3]	0.0006346	0.0000***	− 0.0003748	0.0000***
[1, 5]	0.0009162	0.0000***	− 0.0004542	0.0001***
[1, 10]	0.0006894	0.0090***	0.0001264	0.2119
[1, 20]	0.0003979	0.1151	0.0000321	0.4374

*P < 0.1.

**P < 0.05.

***P < 0.01.

**Table 5:** Significance test results of hydrological dimension.

Time window	2000–2009		2010–2019	
	MCAR	P	MCAR	P
[− 10, − 1]	0.0001204	0.2357	0.0012784	0.0000***
[− 5, − 1]	− 0.0024899	0.0000***	− 0.0016269	0.0000***
[− 3, − 1]	− 0.0007308	0.0000***	− 0.0014368	0.0000***
[1, 3]	− 0.000653	0.0001***	− 0.0002985	0.0076***
[1, 5]	− 0.0000815	0.3230	− 0.0002857	0.0230**
[1, 10]	0.0008674	0.0001***	− 0.0000519	0.3819
[1, 20]	0.0016232	0.0000***	− 0.0011271	0.0000***

*P < 0.1.

**P < 0.05.

***P < 0.01.

**Table 6:** Significance test results of meteorological dimension.

Time window	2000–2009		2010–2019	
	MCAR	P	MCAR	P
[− 10, − 1]	0.0035956	0.0000***	− 0.0005223	0.0009***
[− 5, − 1]	0.0008252	0.0162**	− 0.0005553	0.0000***
[− 3, − 1]	− 0.0004599	0.0669*	− 0.0007627	0.0000***
[1, 3]	0.0002555	0.1638	− 0.0009274	0.0000***
[1, 5]	0.0008757	0.0007***	− 0.0012374	0.0000***
[1, 10]	0.0013802	0.0000***	− 0.0025335	0.0000***
[1, 20]	0.0009991	0.0019***	− 0.0032708	0.0000***

*P < 0.1.

**P < 0.05.

***P < 0.01.

#### Biological dimension

Table 2 indicates that the average cumulative excess returns of the sample stocks are already responding to the disaster events of this dimension from 10 days before the outbreak of the biological events and gradually increasing. The average cumulative excess returns of the sample stocks are presented in (see Supplementary Fig. S1 online). It can be observed that the shock to U.S. stock returns prior to the onset of biological events is reflected by an increase and subsequent decrease. Conversely, the shock returns become positive to negative following the occurrence of biological events. Consequently, the U.S. stock returns exhibit both anticipated effects and unanticipated effects due to the biological dimension of climate change. The empirical results demonstrate that prior to the emergence of the biological disaster, the U.S. stock market has identified the potential impact of the disaster on the financial market and adjusted its expectations in line with the evolving situation. Following the onset of the biological disaster, the U.S. stock market responded promptly to the evolving information, resulting in a decline in yields and a sustained effect due to the prolonged duration of the disaster.

#### Climatic dimension

Table 3 illustrates that the average cumulative excess returns of the sample stocks over the period 2000–2009 respond with the highest confidence level to the shock from 3 days before a climatic disaster. Over the period 2010–2019, the average cumulative excess returns of the sample stocks have responded to the dimensional shock from 10 days before a climate disaster, which is significant at the 99% confidence level for all time windows, with the exception of the average cumulative excess returns of U.S. stocks over the period 3 days after a climatic disaster. (See Supplementary Fig. S2 online) illustrates that the shock to U.S. stock returns is reflected as a positive effect before climatic disasters and a volatile decrease after climate disasters over the period 2000–2009. The impact of U.S. stock returns on the climatic dimension of climate change can be described as both short-term and shock effects. During the period of 2010–2019, the impact on U.S. stock returns before a climatic disaster is characterised by a negative effect, while after a climatic disaster it is characterised by a volatile increase. Consequently, U.S. stock returns exert an expected effect and a medium-term and long-term shock effect on the climatic dimension of climate change. The empirical results indicate that prior to the occurrence of a climatic disaster, the U.S. stock market relies on weather forecasts and other sources of information to inform its decisions, and subsequently reflects this information in the stock market. Conversely, following the occurrence of a climatic disaster, the U.S. stock market processes the new information in an anticipatory manner, potentially resulting in an underreaction phenomenon and a persistent effect. A review of the empirical results over two time periods indicates that the impact of the climatic dimension of climate change on U.S. stock returns is time-varying. The development of weather forecasting technology and the stock market has enabled a more nuanced understanding of the impact of this dimension of climate change on U.S. stock yields. This impact is now understood to have a longer time span, greater volatility, and greater significance.

#### Geological dimension

Table 4 illustrates that during the period 2000–2009, the average cumulative excess returns of the sample stocks exhibited a significant increase in the 10 days following the occurrence of geological disasters. Conversely, during the period 2010–2019, the average cumulative excess returns of the sample stocks demonstrated a significant decrease in the 5 days following the occurrence of geological disasters. The average cumulative excess return of the sample stocks is summarized by (see Supplementary Fig. S3 online). The average cumulative excess return of the sample stocks after the occurrence of geological disasters in both phases is observed to exhibit an increase and subsequent decrease. In contrast, the U.S. stock returns are found to have a shock effect, while neither an expected effect nor a continuous effect is evident. The empirical evidence indicates that the predictability of the geological shock in climate change is extremely low and varies over time. It can be observed that geological events are always influenced by the scale, scope and degree of loss. Furthermore, the direction of the impact of US stock market returns differs. The duration of the impact of geological shocks on the U.S. stock market is shorter in comparison to other shocks, contingent on the government's capacity to respond to emergencies.

#### Hydrological dimension

Table 5 and (see Supplementary Fig. S4 online) illustrates that during the period 2000–2009, the average cumulative excess returns of the sample stocks exhibited a negative response to the climate change shock, with the impact being observed 5 days prior to the occurrence of the hydrological disaster. However, the impact was not sustained and the average cumulative excess return subsequently recovered, turning from negative to positive 5 days after the hydrological event. The results indicate that there is a short- to medium-term anticipation and shock effect. During the period of 2010–2019, the average cumulative excess returns of the sample stocks have responded to the impact of this dimension from 10 days before the hydrological disaster. Furthermore, from 5 days after the event, there was a fluctuation in the returns. It can be seen that there are two distinct effects: an expected effect and a shock effect. The empirical results indicate that the long-run expected effect of the hydrological dimension of climate change is gradually emerging, the shock effect is weakening, and the impact on U.S. stock returns is time-varying.

#### Meteorological dimension

Table 6 illustrates that the average cumulative excess returns of the sample stocks during the period 2000–2009 have responded to climate change 10 days prior to the meteorological disaster. This response is significant at the 90% confidence level for all time windows, with the exception of the 3 day shock, which is insignificant. Furthermore, the average cumulative excess returns of the sample stocks during the period 2010–2019 are statistically significant at the 99% confidence level for all time windows. (See Supplementary Fig. S5 online), illustrates that during the period 2000–2009, the U.S. stock returns reflect the meteorological dimensional shocks of climate change, with a decrease followed by a slow increase. In contrast, during the period 2010–2019, the U.S. stock returns reflect a persistent negative effect. The effects are both short-term and expected, as well as shock effects, in both periods. The empirical results indicate that the impact of the meteorological dimension of climate change on U.S. stock returns is time-varying. During the period between 2000 and 2009, the U.S. stock market exhibited a tendency to overreact and subsequently adjust gradually, relying on weather forecast information prior to the occurrence of a meteorological disaster. This resulted in a short-term underreaction following the meteorological disaster event. In contrast, between 2010 and 2019, the U.S. stock market demonstrated a high degree of sensitivity to the meteorological dimension of climate change with a long time horizon. In conclusion, the impact of climate change on the U.S. stock market is time-varying.

In summary, the U.S. stock market is more susceptible to the climatic, hydrological, and meteorological dimensions of climate change. The climatic and meteorological dimensions of disaster event shocks have increased in impact, while the hydrological dimension has a relatively flat impact and led to a lower stock return volatility. One potential explanation for the relatively smooth impact of hydrological dimensions on stock returns is that water resource issues have a more indirect and frequent impact on firms than sudden climate events such as meteorological hazards. The market is already accustomed to facing these challenges. Alternatively, the market may have already anticipated and digested these risks to some extent. The geological dimension tends to be more of a short-term shock, with a much smaller overall impact, while the biological dimension shocks are more persistent. However, further analysis of this dimension is limited by the lack of data after 2010.

## Discussion

According to the results, the five dimensions of climate change have a significant impact on US equity yields. Among them, the impact of climate dimension, meteorological dimension and hydrological dimension is intensified than before 2010. Meanwhile, the geological dimension impact has high intensity, short duration and large negative impact, showing the characteristics of long duration and wide influence range. Based on the above conclusions, this paper suggests that investors should take climate risk as the necessary basis for decision-making when choosing investment targets, and formulate investment strategies based on the potential risks of the investment portfolio based on each dimension of climate change. Investors should pay more attention to the information on drought, wildfire, hurricane, and flood and adjust their investment positions. During the investment process, assess the industry and geological events, and make investment plans; improve the event analysis ability and adjust the long-term and short-term investment strategies to avoid herd effect. The government should improve the information disclosure mechanism, develop weather forecast, earthquake early warning and other technologies, improve the infectious disease prevention system, support and encourage enterprises to formulate climate change mitigation and response measures, promote carbon reduction and carbon neutrality, and solve the impact of climate change on the US stock market from the source; Meanwhile, the government should improve the infrastructure construction, maintain the order of financial market, enhance the capacity of the financial market to withstand climate risks, minimize the economic and social damage caused by climate change and its impact on the stock market, and avoid panic selling by investors due to extreme climate events. Listed companies should enhance their climate resilience, comprehensively assess their climate risks according to their industry and location, make contingency plans, and improve their ability to respond to the “black swan” events of climate change. Financial institutions should develop diversified financial derivatives for climate change, such as catastrophe insurance and options and bonds related to extreme weather, to hedge against climate risks and enhance the stability of the financial system.

## Methods

### Event study method

With regard to climate change, scholars at home and abroad have conducted extensive research. Climate change is evidenced by an increase in the frequency, scale, and other characteristics of natural disasters. In the existing literature, the event study method is a widely accepted approach. In light of this, this paper employs the event study method to investigate the impact of climate change on U.S. stock returns.

The event study model is a powerful econometric tool for estimating dynamic processing effects. One of its most attractive features is that it creates a built-in graphical summary of the results. The event study method in economics begins with financial applications and the study of the impact of stock segmentation on trading activities, data payment rates, and market returns. The event study model consists of the multiplication of a core explanatory variable and time, several control variables and two-way fixed effects, and random disturbance terms. The core explanatory variable of this paper is the stock return, and the time is the date of the climate change event.

### Method implementation

#### Determine the event date and time window

The date of occurrence of a climate change event is defined as event day.

The event day is the day on which the climate change event occurs. In the event that the day on which the climate change event occurs is not a trading day, the first trading day following the climate change event is considered to be the event day, that is, day 0, which is denoted as $t = 0$. The first trading day before the event is $t = - 1$, the second trading day before the event is $t = - 2$, and so on; the first trading day after the event is $t = 1$, the second trading day after the event is $t = 2$, and so on. The time window is the study period during which this event has an impact, and in this paper we choose [− 5, 5] as basic time window for the event study.

#### Measuring stock returns

1 $$ Rit = \frac{closet = 0 - closet = - 1}{{closet = - 1}} $$

where: $closet = 0$ denotes the closing price of the stock on the day the stock return is calculated; $closet = - 1$ denotes the closing price of the stock the day before the stock return is calculated.

#### Measurement of normal rate of return

The selected sample companies are listed companies that are constituents of the NASDAQ 100 index. As such, the NASDAQ 100 index returns can be considered as sufficiently diversified market returns to serve as normalised returns for the sample companies.

#### Measuring excess returns

2 $$ ARit = Rit{-\!\!-}Rmt $$

where: $ARit$ denotes the excess return of the $i{\text{th}}$ th sample on the $t$ th day;$Rit$ denotes the stock return of the $i$ th sample on the $t$ th day;$R{\text{m}}t$ denotes the return of the NASDAQ 100 on the $t$ th day.

#### Measuring cumulative excess returns

3 $$ CARit = \sum\limits_{t = t1}^{t2} {ARit} $$

where: $CARit$ represents the cumulative excess return of $t$ for the first $i$ sample.

#### Measuring average cumulative excess return

4 $$ MCARit = \frac{1}{N}\sum\limits_{i = 1}^{N} {ARit} $$

where: $MCARit$ denotes the average cumulative excess return of all samples on day $t$.

#### Event study model

5 $$ MCARTit = \lambda it \cdot ARit \cdot t + \theta it \cdot controlsit + \alpha i + \beta t + \varepsilon it $$

where: $\lambda it$ and $\theta it$ denote coefficients, $\alpha i$ $\beta t$ $\varepsilon it$ denote two-way fixed effects, and random disturbance terms.

### Data selection

The data of climate change events and related indicators selected in this paper for the years 2000–2019, totaling 526, are from the EM-DAT database and are compared with the data from the Global Platform for Disaster Data to confirm that the two are consistent and the data from EM-DAT are more comprehensive. Following the categorisation of the EM-DAT database, as shown in Table 7, this paper classifies climate change events into biological, climatic, geological, hydrological and meteorological dimensions. This paper wants to study the impact on the US stock market in different periods of climate change, and climate change is a slow process, so this study takes 10 years as the period, and 2000–2009 and 2010–2019 are selected as two comparison periods.

**Table 7:** Topology of natural disasters.

Main types of natural disasters	Natural disaster subtypes
Biological disaster	Epidemic
	A swarm of insects attacking
	Animal accidents
Climatic disaster	Drought
	Glacial lake outburst
	Wild fire
Geological disaster	Earthquake
	Plate movement
	Volcanic eruption
Hydrological disaster	Fog
	Floods
	Landslide avalanche
	Wave action
Meteorological disaster	Tropical storm
	Extreme temperatures

Since the subject of this paper is the US stock market, it is important to choose a representative stock index. The impacts of climate change can be broadly divided into physical and transformational impacts, and this paper focuses mainly on the physical impacts. The NASDAQ 100 index excludes financial industries, thereby eliminating the superposition of the impact of its transformational impact. Furthermore, the index is concentrated in the high-tech industry, whose sample capacity is moderate, and the sensitivity of the external shock is strong. Consequently, this paper selects the NASDAQ 100 index as the research sample. The data were obtained from historical data on investing, which excluded samples that were suspended on the day of the climate change event, samples without continuous trading quotations within the event window, and samples with incomplete data. After screening, 93 constituents of the NASDAQ 100 remained.

### Supplementary Information

Supplementary Information.

## Data Availability

The datasets generated during and/or analysed during the current study are available from the corresponding author upon reasonable request. The relevant URLs are as follows, and the relevant spreadsheets can be found in the Supplementary file. EM-DAT database: https://public.emdat.be/data (Please open it with the international network). Stocks data: https://cn.investing.com/indices/nq-100-components
