# Supplementary material for: A study of impact of climate change on the U.S. stock market as exemplified by the NASDAQ 100 index constituents
Source: Sci Rep. 2024 Jul 5;14:15468. doi: 10.1038/s41598-024-66109-7 (PMC11226447; doi:10.1038/s41598-024-66109-7)
Supplement: Supplementary file 1 — Supplementary Information. [file 41598_2024_66109_MOESM1_ESM.docx]

**Supplementary**

**Title:** A Study of Impact of Climate Change On The U.S. Stock Market As Exemplified By The NASDAQ 100 Index Constituents

**Authors:** Cunpu Li Yingjun Liu Lishuo Pan

**Supplementary Spreadsheets**

EM-DAT database: [Public EM-DAT platform (emdat.be)](https://public.emdat.be/data) (Please open it with the international network)

Stocks data: <https://cn.investing.com/indices/nq-100-components>

**Accession codes**

**Supplementary Figures**

**Fig. S****1** Values of MCAR for different time windows of biological dimension.

The figure generated by EXCEL 2016. The blue line indicates the changes in MCAR values before and after climate change events from 2000 to 2009.

**Fig. S****2** Values of MCAR for different time windows of climatic dimension.

The figure generated by EXCEL 2016. The blue line indicates the changes in MCAR values before and after climate change events from 2000 to 2009；the orange line indicates the changes from 2010 to 2019；gray lines indicate non-significant MCAR values corresponding to windows.

**Fig. S****3** Values of MCAR for different time windows of geological dimension.

The figure generated by EXCEL 2016. The blue line indicates the changes in MCAR values before and after climate change events from 2000 to 2009；the orange line indicates the changes from 2010 to 2019；gray lines indicate non-significant MCAR values corresponding to windows.

**Fig. S****4** Values of MCAR for different time windows of hydrological dimension.

The figure generated by EXCEL 2016. The blue line indicates the changes in MCAR values before and after climate change events from 2000 to 2009；the orange line indicates the changes from 2010 to 2019；gray lines indicate non-significant MCAR values corresponding to windows.

**Fig. S****5** Values of MCAR for different time windows of meteorological dimension.

The figure generated by EXCEL 2016. The blue line indicates the changes in MCAR values before and after climate change events from 2000 to 2009；the orange line indicates the changes from 2010 to 2019；gray lines indicate non-significant MCAR values corresponding to windows.
